# Structural-Controlled Synthesis of Highly Efficient Visible Light TiO_2_ Photocatalyst via One-Step Single-Mode Microwave Assisted Reaction

**DOI:** 10.1038/s41598-019-41465-x

**Published:** 2019-03-20

**Authors:** Kunihiko Kato, Yunzi Xin, Takashi Shirai

**Affiliations:** Advanced Ceramics Research Center, Nagoya Institute of Technology, Gokiso-cho, Showa-ku, Nagoya, Aichi 466-8555 Japan

## Abstract

TiO_2_ with different chemical structures are successfully synthesized via a one-step single-mode magnetic microwave (SMMW) assisted process, during where Ti selectively oxidizes in magnetic field under Ar-O_2_ mixed atmosphere. The chemical state and band structure of the as-prepared TiO_2_ are well-controlled by changing the volume fraction of O_2_ (φO_2_) during SMMW synthesis. Ti^3+^ self-doped TiO_2_ (TiO_2−x_, 0 < x < 2) is synthesized under lower φO_2_, while TiO_2_ with specific core/shell structure (TiO_2+y_ core/TiO_2−x_-TiO_2+z_ shell) is observed under higher φO_2_. The as-synthesized TiO_2_ with controlled structures show sufficient light absorption in visible region and quite narrow bandgap (2.05 eV∼), whose value can be also tuned by φO_2_ during SMMW synthesis. In addition, the synthesized TiO_2_ exhibits highly efficient photocatalytic performance towards the degradation of Rhodamine B under UV and visible light irradiation. The formation mechanism for different structural TiO_2_ can be attributed to the specific rapid heating and cooling dynamics induced by SMMW irradiation.

## Introduction

TiO_2_ photocatalysis have been widely utilized due to its physical and chemical stability, high photocatalytic activity, and nontoxicity^1,2^. However, the wide bandgap (3.0–3.2 eV) of TiO_2_ seriously limits its absorption wavelength and photocatalytic performance only in UV light region. Numerous efforts have been paid for enhancing photocatalytic performance of TiO_2_ in visible light region by inducing doped level from metals^3,4^ or nonmetals^5–8^. However, the element doping may cause thermal or crystal instability and an increase on carrier trapping, which may decrease the photocatalytic efficiency^9^. In recent years, Ti^3+^ self-doped TiO_2_ has attracted much interest, since surface energy level induced by Ti^3+^ and oxygen vacancies can improve visible-light absorption and results in high photocatalytic performance^10–15^.

In our previous paper, we initially reported a novel single-mode magnetic microwave (SMMW) assisted one-step synthesis of Ti^3+^ self-doped TiO_2_^16^. Upon the one-step irradiation of SMMW in pure oxygen atmosphere, Ti target oxidizes in tens of second reaction by rapid temperature change. Such specific heating process can be attributed to the drastic change of MW absorbing property accompanied with changes of chemical state in obtained material. Here in the present research, TiO_2_ with well-controlled chemical states and band structures were selectively prepared by altering the oxygen fraction (φO_2_) in an Ar-O_2_ mixed atmosphere during SMMW synthesis. Ti^3+^ self-doped TiO_2_ (TiO_2−x_, 0 < x < 2) is synthesized under lower φO_2_, while TiO_2_ with specific core/shell (TiO_2+y_ core/TiO_2−x_-TiO_2+z_ shell) structure is observed under higher φO_2_. The structure-controlled TiO_2_ show sufficient light absorption in visible region with narrower bandgap (2.05 eV∼), whose value can be also well tuned by φO_2_. Meanwhile, the synthesized TiO_2_ show superior photocatalytic performance to commercial TiO_2_ in degradation of Rhodamine B (RhB) under both UV and visible light irradiation. The formation mechanism for different structural TiO_2_ is clarified based on systemically investigation on crystallinity analysis with X-ray diffraction (XRD) patter and Raman spectroscopy, confirmation of chemical state by X-ray photoelectric spectroscopy (XPS), characterization of electrical band structures with UV-visible and photoluminescence (PL) spectroscopy. The SMMW assisted synthesis process described in this paper provides new strategy for the development of functional metal oxides with well-controlled chemical structures and specific properties.

## Results and Discussion

**Figure 1:**
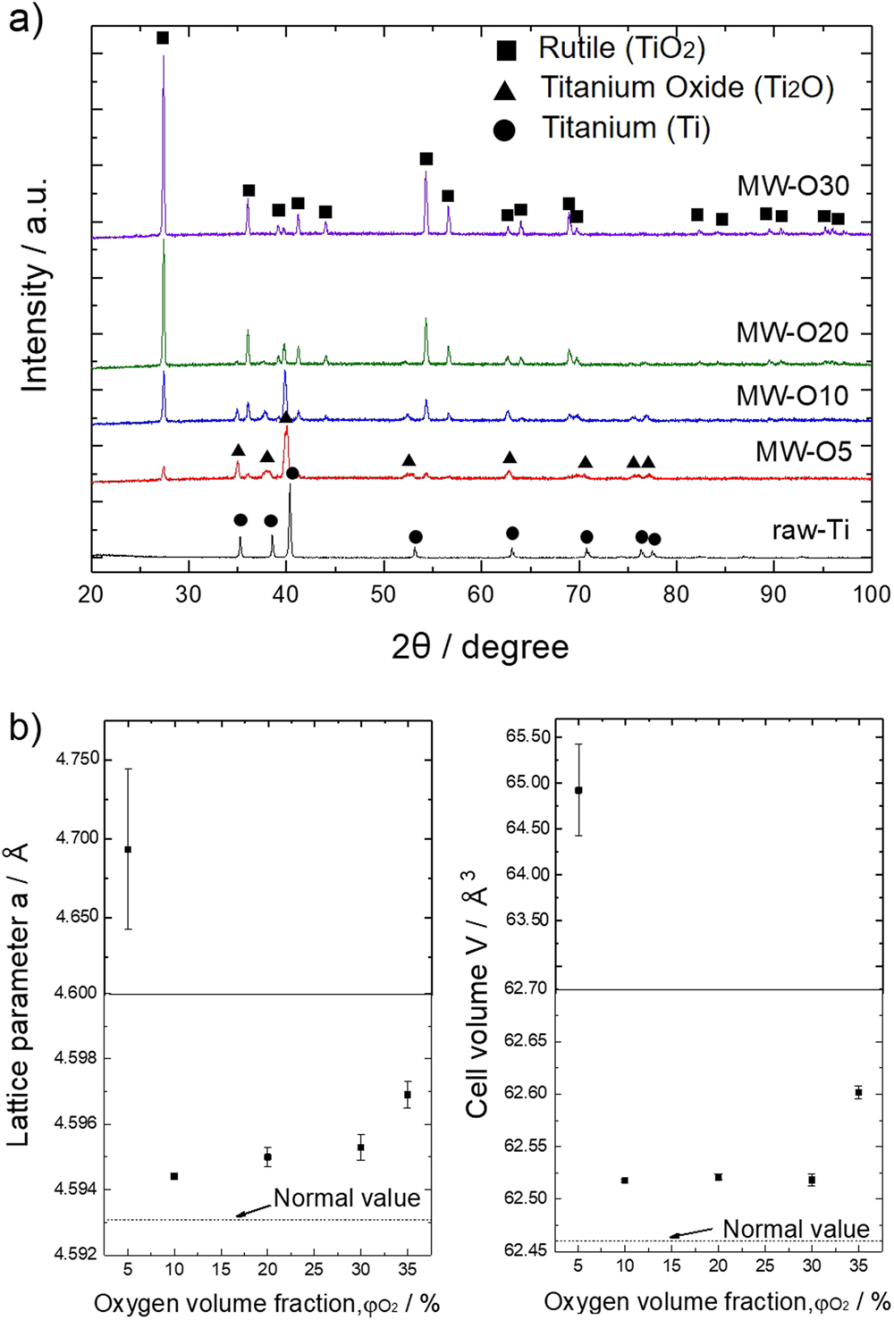
(**a**) XRD patterns of raw-Ti and as-prepared TiO_2_. (**b**) Changes of the a-axis and cell volume of rutile TiO_2_ synthesized under various atmosphere; the dashed lines indicate the value of rutile TiO_2_ phase (JCPDS-ICDD card No. 01-084-1283) as reference.

Figure 1a shows XRD pattern of the as-synthesized TiO_2_ samples. In the case of TiO_2_ prepared by MW irradiation under lower φO_2_ (MW-O5/MW-O10/MW-O20), two sets of diffraction peaks can be observed, corresponding to rutile TiO_2_ and Ti_2_O. With increasing of φO_2_, the intensity of rutile diffraction peaks increased while Ti_2_O peaks was disappeared, clearly demonstrates the progressive oxidation of target Ti. In addition, Figure 1b presents the change of lattice parameter of rutile TiO_2_ as a function of φO_2_. Interestingly, the lattice constant of a-axis and cell volume (V) significantly varied with φO_2_, taking the minimum value in the sample MW-O10. In the case of sample synthesized at the lowest φO_2_ (MW-O5), the a-axis lattice constant and V show the largest value. For samples synthesized under higher φO_2_ (MW-O10, MW-O20, MW-O30), the a-axis lattice constant and V are much smaller than MW-O5 and these values are gradually increased as φO_2_ increased. It has been reported that when oxygen vacancy is induced in TiO_2_, the Ti-Ti and Ti-O bonds are strongly relaxed and the nearest-neighbor Ti atoms move outward from the vacancy along a-axial, leading to the expanding of lattice constant of a-axis^17^. Thus, the expended large a-axis constant and V in sample MW-O5 can be attributed to the induce of oxygen defects. On the other hand, in the case of TiO_2_ possessing excessive oxygen atom, Ti-O bond is slightly relaxed because interstitial O atom repulse against lattice O atom, and coordinate with nearest-neighbor Ti atom, whose phenomenon seems to cause a slightly expanded lattice. It can be suggested that the TiO_2_ prepared under higher φO_2_ consists of both TiO_2−x_ and TiO_2+y_ states with regular arrangement of oxygen vacancy and excessive O atom, respectively.

**Figure 2:**
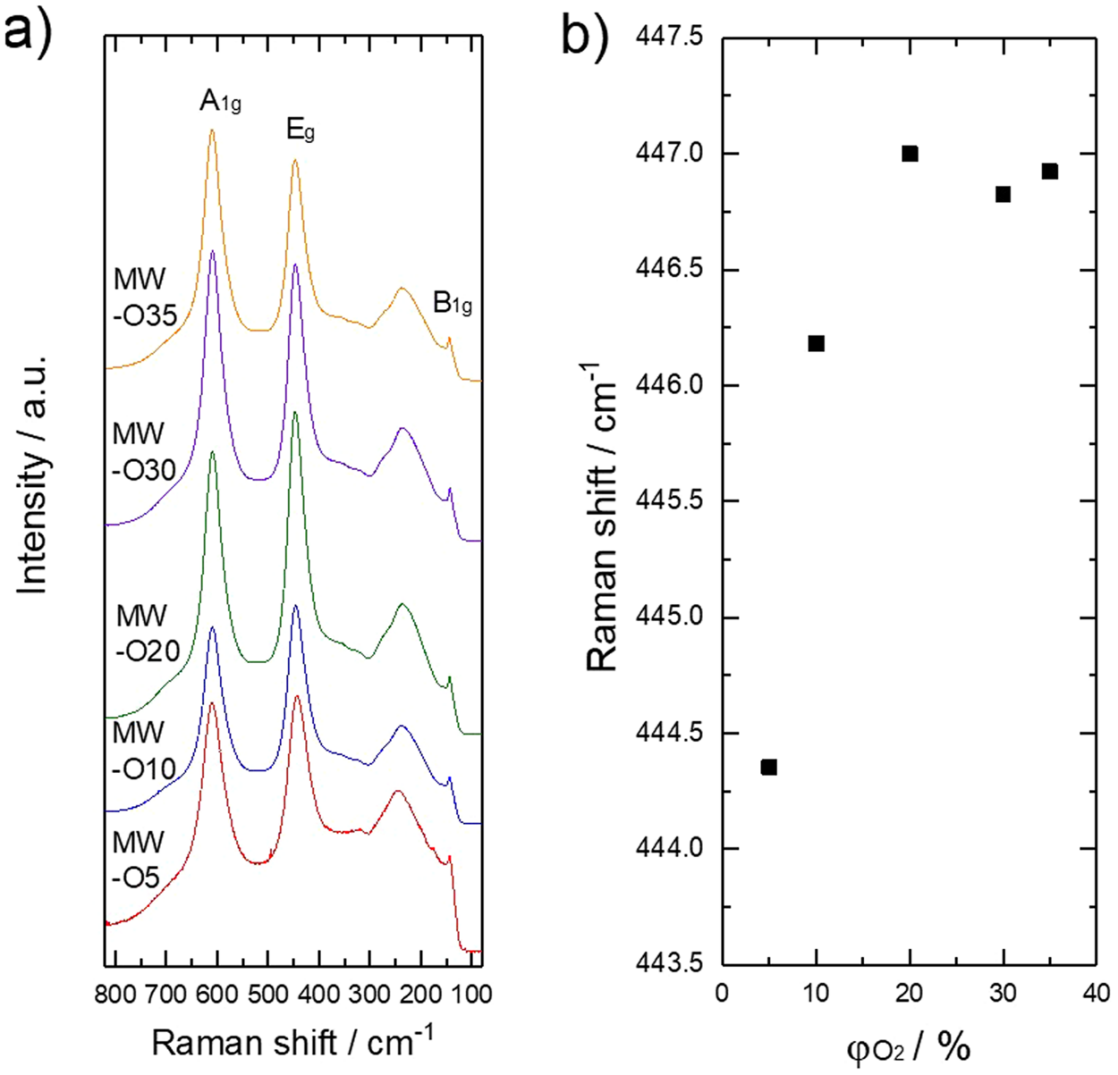
(**a**) Raman spectra of as-prepared TiO_2_. (**b**) The Changes of the Raman shift of rutile TiO_2_ Raman active modes (E_g_ mode).

Figure 2 shows Raman spectra of synthesized samples, summarized Raman shift of E_g_ mode of TiO_2_ as a function of φO_2_. As a result, characteristic peaks which are attributed Raman active modes of rutile crystal phase are observed in the spectra^18,19^. The peak of E_g_ mode is red-shifted with decrease of φO_2_. The red-shifting of E_g_ can be attributed to the inducing of oxygen vacancy defects since it has been reported that E_g_ mode is sensitive against oxidation state of rutile TiO_2_ in Raman spectra, which would be significantly red-shifted due to formation of oxygen vacancy defects in TiO_2_^20,21^.

**Figure 3:**
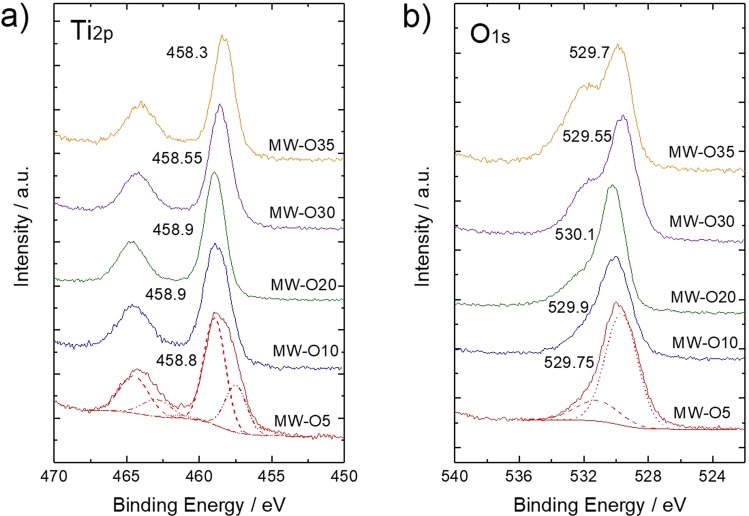
XPS spectra of core levels (**a**) Ti_2p_ and (**b**) O_1s_ related to as-prepared TiO_2_. The number indicate the center value of peak top.

**Figure 4:**
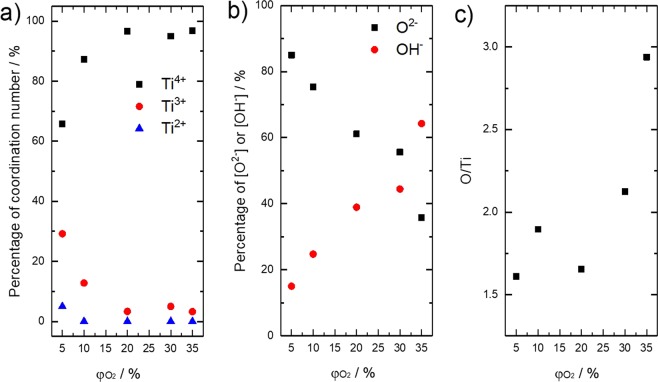
(**a**) Percentage of coordination number of titanium from XPS spectra of core levels Ti_2p_. (**b**) Percentage of O^2−^ and OH^−^ from XPS spectra of core levels O_1s_. (**c**) Composition of as-prepared TiO_2_.

The chemical structure of TiO_2_ were furtherly investigated by confirming of surface chemical bonding sates via XPS, as shown in Figure 3. In the spectra of Ti_2p_ orbital (Figure 3a), the peaks varied from 458.8 to 458.9, 458.9, 458.6, and 458.3 eV with increasing of φO_2_. These peaks are fitted by decomposed peaks at 458.8, 457.1 and 455.5 eV. The peak at 458.8 eV can be assigned as Ti^4+^ state in TiO_2_, and the peaks at 457.1 and 455.5 eV are assigned as Ti^3+^ and Ti^2+^, whose binding energy is 1.7 and 3.4 eV lower than that of Ti^4+^, respectively^22,23^. The peak components of Ti^4+^, Ti^3+^ and Ti^2+^ as a function of φO_2_ are summarized in Figure 4a. It can be clearly demonstrated that Ti^3+^ and Ti^2+^ components decrease as φO_2_ decreases, while Ti^4+^ component increases as φO_2_ increases. As for O_1s_ spectra as shown in Figure 3b, peaks vary as 529.75, 529.9, 530.1, 529.55 and 529.7 eV for different samples and a shoulder peak appears at about 532.0 eV with φO_2_ increases. These peaks at 530.1 and 531.7 eV are assigned as O^2−^ (Ti-O) and OH^−^, respectively^23–25^. According to the results summarized in Figure 4b, the concentration of OH group increases with increase of φO_2_. It has been reported that oxygen vacancy may induce dissociation of H_2_O, then chemisorption of OH group takes place on the surface of TiO_2_^1,26^. Thus, the obtained results indicate that the TiO_2_ synthesized under high φO_2_ contain high concentration of oxygen vacancy. Furthermore, this oxygen vacancy would induce strong relaxation between Ti-O bonding as being already described, leading to chemical shift of Ti^4+^ peak center in Ti_2p_ level toward lower binding energy. Figure 4c shows surface chemical composition of as-prepared samples estimated by peak area ratio in O_1s_ to Ti_2p_. It demonstrates that excessive oxygen exists in TiO_2_ lattice synthesized under high φO_2_. Furthermore, the ratio of O/Ti was likely to decrease with decrease of φO_2_. This result might be not consistent with the fact that TiO_2_ synthesized under high φO_2_ contains high concentration of oxygen vacancy. Thus, here we suggest that the obtained TiO_2_ may possess quite specific chemical structure, which oxygen vacancy and interstitial oxygen atom coexist in TiO_2_ crystal on top of particle surface.

**Figure 5:**
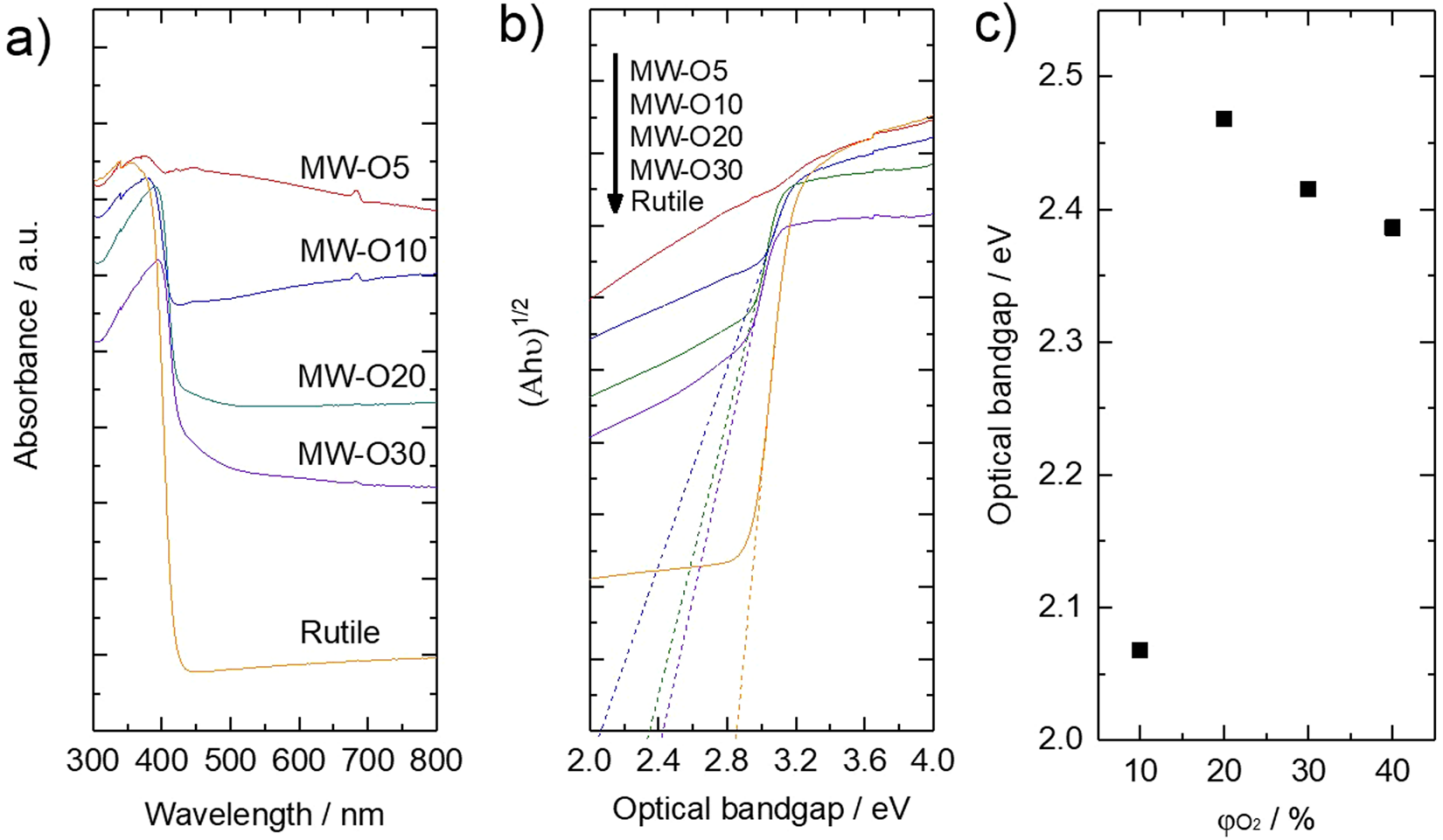
(**a**) UV-vis spectra of as-prepared TiO_2_ and commercial rutile TiO_2_. (**b**) Tauc plot. (**c**) Optical bandgap.

**Figure 6:**
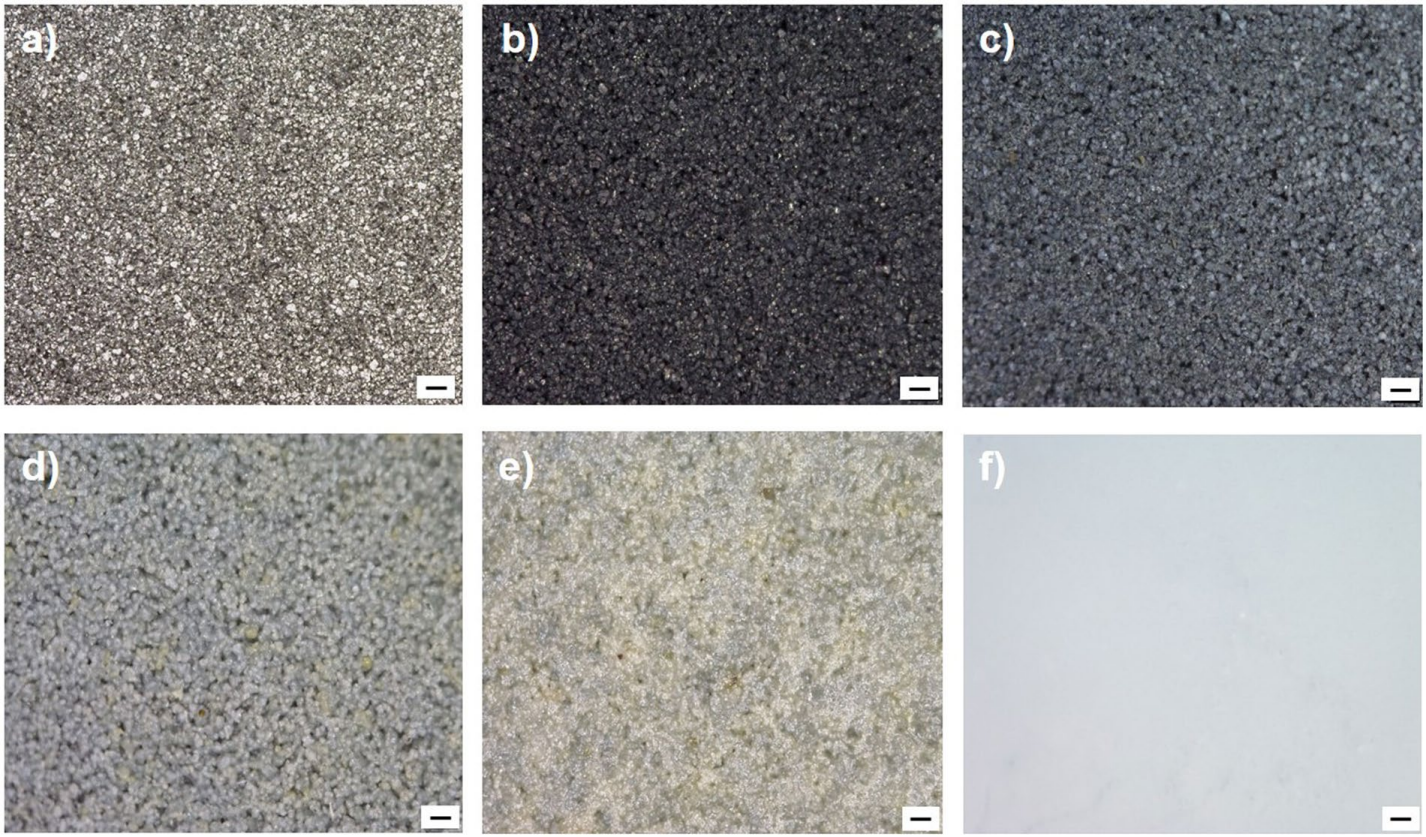
CCD images of raw Ti and as-prepared TiO2. (**a**) raw Ti, (**b**) MW-O5, (**c**) MW-O10, (**d**) MW-O20, (**e**) MW-O30, (**f**) P-25. The scale bar represents 100 μm.

The electrical absorption spectra of as-prepared sample were characterized as shown in Figure 5a. The as-prepared TiO_2_ exhibit superior light absorption in visible region to compare with commercial rutile TiO_2_. In addition, the shoulder peak from 400 to 500 nm gradually appear with increase of φO_2_ due to formation of donor level between valence band (VB) and conduction band (CB) of TiO_2_ caused by interstitial oxygen atom in lattice^27^. Furthermore, the optical bandgaps are calculated by Tauc plot in Figure 5b and the bandgap values are summarized as Figure 5c. As a result, the maximum value of bandgap is observed in sample MW-O20, whose value becomes significantly smaller with lower φO_2_, while becomes slightly narrower with higher φO_2_ as shown. As bandgap of sample MW-O10 gives value of 2.07 eV, it is in good agreement with TiO_2_ where Ti^3+^ and oxygen vacancy introduce localized states at 0.75–1.18 eV below the CB minimum in the case of TiO_2−x_^13^. On the other hand, although interstitial oxygen atom localized the state about 0.2~0.3 eV above the VB maximum in the existence of excessive oxygen in TiO_2_ in general^27^, the obtained bandgap of TiO_2_ synthesized under high φO_2_ (MW-O20, MW-O30 and MW-O35) were much narrower. According to the CCD photograph of synthesized TiO_2_ as shown in Figure 6, the color of samples changed from metal gray to black, grey, ash grey and yellowish grey with different φO_2_. It is known that TiO_2+x_ performs yellowish, on the contrast, the color of TiO_2_ with oxygen vacancy become grey or black because trapped electron in oxygen vacancy act as color center. Thus, TiO_2_ synthesized under high φO_2_, especially MW-O20, MW-O30 and MW-O35 have complex impurity level caused by oxygen vacancy and interstitial oxygen atom, resulting in formation of narrow bandgap.

**Figure 7:**
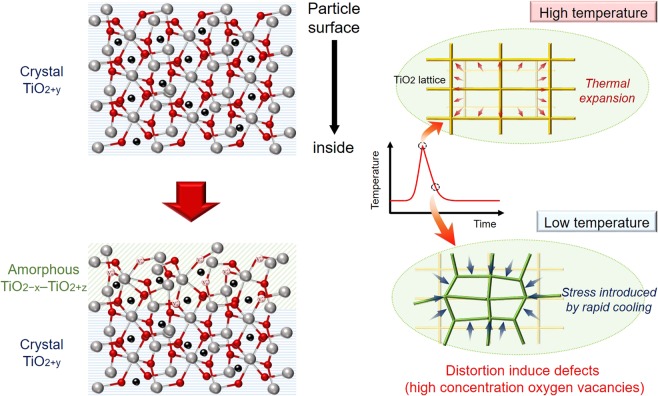
Mechanism of formation of TiO_2+y _core/TiO_2-x_ – TiO_2+z_ shell structure in single-mode MW heating.

According to XRD pattern, Raman spectra and electrical absorption spectra results, it can be concluded that TiO_2_ synthesized under high φO_2_ exhibit specific TiO_2+y_/TiO_2−x_-TiO_2+z_ core/shell structure. Here, the formation mechanism of such core/shell TiO_2_ during SMMW synthesis is concluded in Figure 7. We suggest that the specific heat history in MW heating is one of key points to form the above specific particle structure. During MW synthesis under high φO_2_ system, TiO_2+y_ would be firstly formed at high temperature, and rapidly cooled to room temperature immediately after formation of TiO_2_ due to lowering magnetic MW absorption, accompanying with large shrink of expanded lattice and introduction of high stress on outermost particle surface. Then the formed strain induces high concentration of defect, especially oxygen vacancy. It has been reported that rapid cooling after formation of oxide from metal target generates high concentration of defects on particle surface^28,29^. Furthermore, there are large densities of oxygen vacancies in TiO_2_ which have plastic strain induced by two anvils^30^. Therefore, amorphous phase of TiO_2−x_-TiO_2+z_ formed on TiO_2+y_, resulting in formation of the specific core-shell structure. On the other hand, during MW synthesis under low φO_2_ system, Ti^3+^ doped TiO_2_ would be formed by the selective oxidation of Ti target in H-field throughout rapid heating and short reaction time^14^.

**Figure 8:**
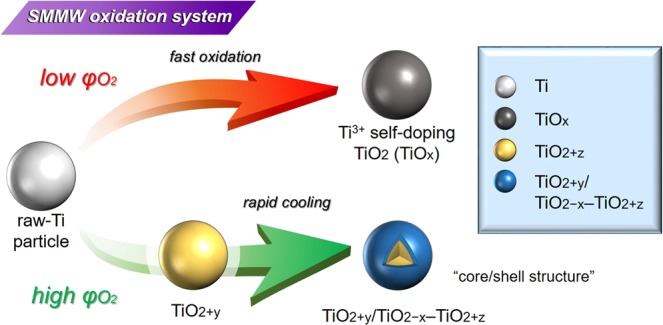
Mechanism of formation of nonstoichiometric TiO_2_ by controlling atmosphere in MW heating.

We summarized the structure controlling mechanism for TiO_2_ during SMMW reaction under different φO_2_ as Figure 8. In the case of SMMW synthesis under low φO_2_, Ti^3+^ self-doping TiO_2_ is formed from Ti particle due to fast oxidation. TiO_2_ synthesized under low φO_2_ contains high Ti^3+^ concentration due to specific heat history through MW reaction. In this case, thermal non-equilibrium reaction lead to uncompleted oxidation of Ti and crystallization of TiO_2_, resulting in formation of nonstoichiometric TiO_2_. Under high φO_2_, oxygen deficient TiO_2_ could be formed by rapid cooling due to introduction of high stress on top surface though the formation of oxygen excessive TiO_2_ at first. In the MW heating process, we control kind of introduced defect, Ti^3+^ and oxygen vacancy and specific chemical structure.

**Figure 9:**
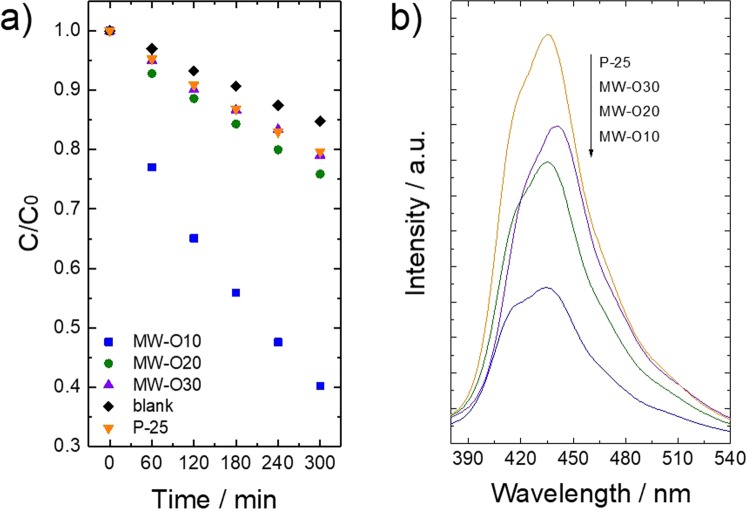
(**a**) Photocatalytic activity towards the degradation of Rhodamine B under irradiation of visible light for constant time (C/C_0_ are the percentage photodegradation of RhB), (**b**) PL spectra of prepared TiO_2_.

As an application of the prepared TiO_2_ with well-controlled structure, the photocatalytic activity towards the degradation of RhB under visible irradiation was examined, whose results are summarized in Figure 9(a). The TiO_2_ synthesized in low φO_2_ (MW-10) exhibits excellent photocatalytic activity under visible light, which can be attributed to the increased light absorption as shown by Figure 5. It worth noting that our synthesized TiO_2_, which possesses a mainly rutile phase and micro-ordered size show even better photocatalytic activity than the nano-ordered P25. The TiO_2_ prepared under high low φO_2_ (MW-20, MW-30) with specific core-shell structure show relative lower photocatalytic activity since high concentration of oxygen vacancies causes recombination of photo-excited carriers. In order to confirm the separation and recombination efficiency of photo-excited charge carriers, PL spectra is measured as Figure 9b. In general, the higher the recombination rate is, the stronger the PL peak intensity is^14,31^. As a result, the as-prepared TiO_2_ exhibited lower recombination rate than commercial TiO_2_, since the PL peak intensity decreases. In this case, the well separated electron and hole carriers transfer into the localized level of Ti^3+^ and VB respectively, leading to sufficient generation of O_2_· and OH· radicals from the reduction of O_2_ and oxidation reaction of H_2_O^32–35^. Such active radicals finally contribute to photo-degradation of RhB under visible light. It can be expected that as-synthesized TiO_2_ perform high photocatalytic efficiency than commercial TiO_2_ with existence of sufficiently separated photo-excited carriers.

## Conclusions

Structurally well-controlled TiO_2_ are successfully synthesized via one-step SMMW assisted process under Ar and O_2_ mixed atmosphere. Ti^3+^ self-doped TiO_2_ and TiO_2+y_/TiO_2−x_-TiO_2+z_ core/shell TiO_2_ are obtained by altering the volume fraction of O_2_. The synthesized TiO_2_ show sufficient light absorption in visible region and narrow band gap. In addition, superior photocatalytic activity for the photo-degradation of RhB under visible light irradiation is observed for the structure-controlled TiO_2_. Despite a large particle size (micrometer order) and rutile crystal phase, our synthesized TiO_2_ shows even better performance than commercial P-25 nanoparticle. The SMMW assisted synthesis process can provide new strategy for the preparation of functional metal oxides with well-controlled chemical structure and specific properties.

## Methods

### Materials and synthesis of TiOx

Titanium powder (3 N, powder under 45 μm mesh, Kojundo Chemical Laboratory, Japan) was used as raw material, and pelletized by uniaxial press. The pressure of 10 MPa was applied to a pellet of 10 mm in diameter. The TiO_2_ were synthesized by magnetic MW heating using 2.45 GHz single-mode MW applicator for TE_103_ mode. The MW output was fixed at 100 W and Ti pellets were heated up under mixed atmosphere of argon and oxygen, whose volume fraction were controlled as Ar/O_2_ = 100-x/x (x = 5, 10, 20 and 30, which named as MW-O5, MW-O10, MW-O20 and MW-O30, respectively).

### Characterization

The crystal structure of the raw material and as-prepared samples were analyzed by X-ray diffraction (XRD) measurement with Cu-Kα (Ultima IV, Rigaku, Japan). The crystal structure in particle surface of obtained sample was furtherly analyzed by Raman spectra (NRS-3100, Jasco, Japan) were measured. The surface chemical state was investigated by X-ray photoelectrical spectroscopy (XPS; M-prove, SSI, USA) with Al Kαsource (hν = 1486.6 eV). The shift of the binding energy due to relative surface charge-up was corrected using the Au_4f_ level at 83.98 eV and C_1s_ level at 284.8 eV. UV-vis absorbance spectra and photoluminescence (PL) spectra were measured by commercial UV-Vis spectrophotometer (V-7100, Jasco, Japan) and spectrofluorometer (FP-8500, Jasco, Japan) at an excitation wavelength of 350 nm, respectively.

### Photocatalytic degradation of RhB

In photocatalytic experiments, as-prepared TiO_2_ and commercial available TiO_2_ pellets catalyst (P-25, Degussa) were loaded into 20 ml of RhB solution (5 ppm). A 200 W Hg-Xe lamp (LA-310UV, HAYASHI, Japan) and Xe lamp (LA-251Xe, HAYASHI, Japan) were used as UV and visible light source, whose powder density was settled as 1 mW cm^−2^. Prior to irradiation, solutions with samples were left to stand in the dark for at least 180 min to ensure that the surface of photocatalysts were saturated with RhB. The RhB degradation was monitored by measuring the changes of UV-vis absorption spectra at 555 nm.
